# Phytochemistry and insecticidal activity of six local plant extracts against *Sitotroga Cerealella* (Oliv.) (Lepidoptera: Gelechiidae) in stored wheat

**DOI:** 10.1186/s12870-025-07879-8

**Published:** 2025-12-29

**Authors:** Ahmed M. A. Salman, Nessma M. Abdeen, Fatma S. Ahmed

**Affiliations:** 1 Department of Plant Protection, Faculty of Agriculture, Sohag University, Sohag, 82524 Egypt; 2 Department of Economic Entomology and Pesticides, Faculty of Agriculture, Cairo University, Giza, 12613 Egypt

**Keywords:** Angoumois grain moth, Eco-friendly, DPPH-IC_50_, Aspartate aminotransferase, Mechanistic responses, Correlation analysis, Postharvest pest

## Abstract

**Background:**

Botanical extracts are emerging as sustainable, multi-mechanistic alternatives to synthetic fumigants for controlling stored grain pests like the Angoumois grain moth (*Sitotroga cerealella*). This study investigated six locally available plants—*Dysphania ambrosioides* (Mexican tea), *Calotropis procera* (calotrope), *Moringa oleifera* (moringa), *Nerium oleander* (oleander), *Lawsonia inermis* (henna), and *Acacia nilotica* (acacia)—to evaluate their phytochemical composition, antioxidant potential, pesticidal efficacy, grain protection capacity, and impacts on insect physiology.

**Results:**

Phytochemical screening revealed strong variation among extracts, with *A. nilotica* having the highest phenolic content. Quantitative assays confirmed that phenolic levels were closely linked to antioxidant capacity, as reflected by the lowest DPPH-IC_50_ value (59.43 µg mL^− 1^).

Bioassays demonstrated dose-dependent insecticidal effects. Adult mortality was particularly rapid with *D. ambrosioides and N. oleander*, with LT50 ≈ 0.38–0.39 days at 10 mg g-1, whereas emergence reduction exceeded 90% at the highest concentrations across most extracts. *Dysphania ambrosioides* consistently demonstrated the highest efficacy, with emergence suppression of up to 98.26%, minimal grain weight loss (0.69%), and the lowest percentage of insect-damaged grains (3.00%). *Acacia nilotica* and *C. procera* also produced strong protective effects at elevated concentrations, whereas *L. inermis* and *M. oleifera* were generally less effective. Biochemical assays revealed that all the extracts significantly reduced aspartate aminotransferase (AST) levels, whereas the *C. procera* and *L. inermis* extracts increased superoxide dismutase (SOD) and reduced glutathione (GSH) levels, indicating diverse mechanistic responses.

Correlation analysis revealed that phenols were negatively associated with AST and alkaline phosphatase, flavonoids were negatively correlated with SOD, and carbohydrates showed strong negative associations with alanine aminotransferase and SOD, as well as positive associations with grain damage indices.

**Conclusion:**

This study demonstrated that plant extracts, particularly *D. ambrosioides, N. oleander*, and *A. nilotica*, exhibit strong bioefficacy against *S. cerealella*, substantially reducing moth development and grain damage. *M. oleifera* and *L. inermis* provided complementary but weaker protection. The observed phytochemistry–bioefficacy correlations suggest that phenolics underpin both antioxidant strength and enzyme suppression, whereas carbohydrate-rich extracts contribute to weaker performance. Collectively, these findings highlight the potential of local botanicals as scalable, safer alternatives to chemical fumigants, supporting sustainable postharvest pest management strategies.

**Graphical Abstract:**

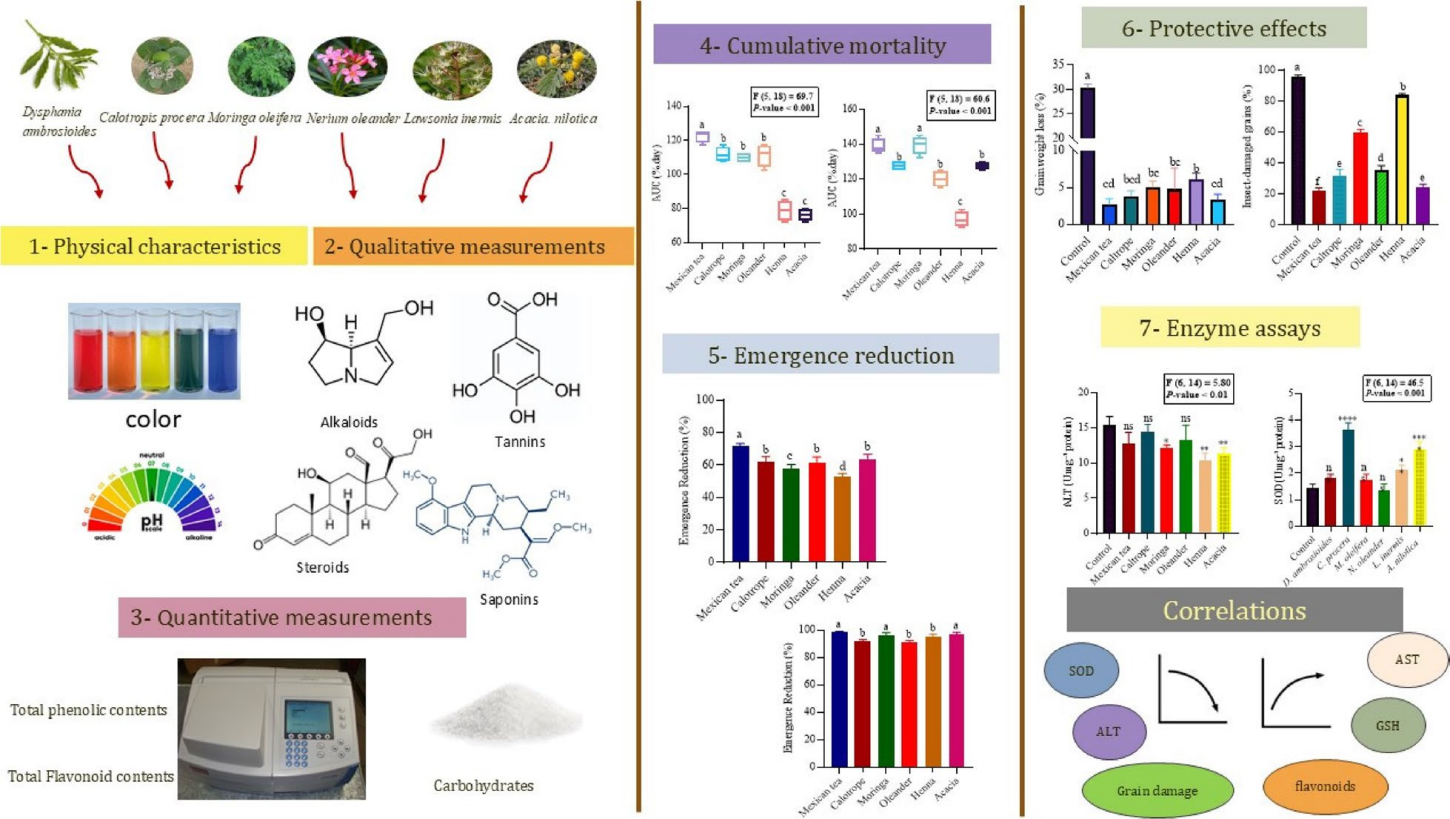

**Supplementary Information:**

The online version contains supplementary material available at 10.1186/s12870-025-07879-8.

## Introduction

Stored grains are highly vulnerable to insect infestations that cause substantial quantitative and qualitative losses worldwide [1]. It is estimated that about 420 million tons of cereals are lost annually because of damage caused by stored-product insects [2]. These losses not only threaten food security but also inflict major economic damage, especially for staple cereals such as wheat, a primary global calorie source [2–4]. Preserving grain quantity and quality during storage is therefore essential for sustainable agriculture and global food supply security.

Among the most damaging pests of stored cereals is the Angoumois grain moth (*Sitotroga cerealella*) (Lepidoptera: Gelechiidae), a cosmopolitan species that predominantly infests whole kernels of wheat, maize, rice, and other grains [5]. The feeding activities of *S. cerealella* larvae cause direct weight loss, nutritional degradation, and contamination with frass, silk webbing, and insect remains, thereby reducing grain quality and marketability. Because immature stages are concealed within kernels, infestations often go undetected until significant damage occurs, making *S. cerealella* particularly difficult to control and a key target for grain protection programs [6, 7].

Traditional control measures have relied heavily on synthetic insecticides (e.g., organophosphates and pyrethroids) and fumigants such as phosphine [8, 9]. Although effective, these chemicals raise serious concerns regarding persistent pesticide residues that threaten consumer health, as well as the widespread development of resistance in stored-grain pests, notably against phosphine [10–12]. The search for safer, sustainable, and effective alternatives to conventional insecticides is therefore an urgent global priority [13].

One promising approach is the use of plant-derived insecticides (botanical extracts).These natural products, long used in traditional pest control, are increasingly recognized as eco-friendly biopesticides [14–16]. Plants synthesize diverse secondary metabolites, such as alkaloids, terpenoids, phenolics, and glycosides, which can act as insecticidal, repellent, or antifeeding agents [17, 18]. Such compounds are biodegradable, often less harmful to non-target organisms, and may offer novel modes of action that mitigate resistance development [19]. Numerous studies have confirmed the efficacy of various plant extracts and essential oils against stored-product insects [2, 19–23], highlighting their potential as sustainable protectants.

Building on this paradigm, the present study evaluates six plant species: *Dysphania ambrosioides* L. (Order: Caryophyllales; Family: Amaranthaceae), *Calotropis procera* (Aiton) (Order: Gentianales; Family: Apocynaceae), *Moringa oleifera* Lam (Order: Brassicales; Family: Moringaceae), *Nerium oleander* L.(Order: Gentianales; Family: Apocynaceae), *Lawsonia inermis* L. (Order: Myrtales; Family: Lythraceae), and *Acacia nilotica* L. (Order: Fabales; Family: Fabaceae), for their potential to control *S. cerealella* in stored wheat. These plants, rich in bioactive phytochemicals, are known in folk medicine or prior pest control studies for their insecticidal potential, yet many remain underexplored in stored-grain protection. For example, *D. ambrosioides* has demonstrated strong fumigant toxicity against storage insects [2], while *C. procera* and *N. oleander* contain potent cardiac glycosides [11, 24]. *Moringa oleifera*,* L. inermis* and *A. nilotica* are also promising due to their saponins, flavonoids, and tannins, compounds associated with insecticidal and repellent properties [25, 26]. Notably, *L. inermis* and *M. oleifera* have rarely been assessed against *S. cerealella*, providing novelty and local relevance to this investigation. We hypothesized that methanolic extracts from these six plants would significantly reduce *S. cerealella* infestation via contact and/or fumigant action.

To evaluate this potential, we assessed their efficacy based on adult mortality and the subsequent emergence of progeny (F1). To move beyond descriptive assays and provide a mechanistic basis for their activity, we first characterized the phytochemical profile of each extract, including total phenolic content, total flavonoid content, and antioxidant activity. Furthermore, we examined the impact of the extracts on key insect physiological parameters and oxidative stress biomarkers to explore potential modes of action. Finally, we performed correlation analyses linking phytochemical constitutes to specific enzyme disruptions and long-term protective effects. This integrative, correlation-based approach aims to clarify which classes of metabolites most strongly underpin adult mortality and emergence suppression, thereby guiding the rational development of botanical formulations tailored for safer, eco-friendly postharvest pest management.

## Materials and methods

### Plant collection and authentication

Six medicinal plants were collected for this study. These included *D. ambrosioides* L. (Mexican tea), *C. procera* (calotrope), *M. oleifera* (moringa), *N. oleander* (oleander), *L. inermis* L. (henna), and *A. nilotica* (acacia). The plants were collected from May to September of 2024 from mature, healthy specimens. The plant materials used were obtained from Sohag University farms, Egypt (26.468733 °N,31.669636 °E). The exceptions included *A. nilotica*, which was collected from El-Balina, Sohag (26.213859 °N,31.993294 °E), and *L. inermis* was collected from Aswan, Egypt (24.472078 °N,32.981454 °E).All the plant samples were authenticated by a qualified taxonomist from the Botany Department, Faculty of Science, Sohag University. Detailed taxonomic information (including order and family), plant parts used, voucher specimen numbers and extraction yields (% w/w) are presented in Table 1.

**Table 1:** Botanical characteristics, plant parts used, voucher specimen numbers and extraction yield of the tested plants

Scientific Name	Order	Family	Plant Part Used	Voucher specimen	Yield (% w/w) ^*^
*Dysphania ambrosioides*	Caryophyllales	Amaranthaceae	Aerial parts	SUH-245-22-05	7.59
*Calotropis procera*	Gentianales	Apocynaceae	Leaves & fruits	SUH-145-20-11	11.57
*Moringa oleifera*	Brassicales	Moringaceae	Leaves & fruits	SUH-72-15-07	10.99
*Nerium oleander*	Gentianales	Apocynaceae	Leaves & flowers	SUH-345-23-12	14.58
*Lawsonia inermis*	Myrtales	Lythraceae	Leaves	ASW-008864	10.59
*Acacia nilotica*	Fabales	Fabaceae	Fruits	SUH-121-21-01	11.99

*The yield of the plant extract (%) was calculated via the following formula: $\:\mathrm{Y}\mathrm{i}\mathrm{e}\mathrm{l}\mathrm{d}\:\left(\%\right)=\frac{\mathrm{M}\mathrm{F}}{\mathrm{M}\mathrm{I}}\times\:100$, where MF is the mass of dried extract after concentration, and MI is the initial mass of the crude plant material

### Preparation of plant extracts

Freshly harvested plant parts were thoroughly washed 2–3 times under running tap water, followed by rinsing with distilled water to eliminate dust, sand, and other surface impurities. The material was then shade-dried at room temperature (23 ± 2 °C) for seven days, with regular turning to ensure even drying. The final moisture content was not measured, but the material was brittle to the touch. Once dried, the plant material was ground via an electric blender (BRAUN^®^, Model JB0123WH, Japan) and passed through a standard laboratory 1 mm mesh sieve (US Standard Sieve No. 18) to obtain a fine powder.

The powder was transferred into airtight containers and stored at 4 °C until extraction. Crude extracts were prepared via cold maceration: 200 g of powdered plant material was soaked in 800 mL of 70% methanol (1:4 powder-to-solvent ratio) in sealed bottles and incubated at 4 °C for 48 h in a cooling shaker incubator (Model: JSS-I^−^100 C) with continuous agitation at 100 rpm. Following incubation, the extracts were vacuum-filtered through Whatman No. 1 filter paper (Maidstone, Kent, UK) via a vacuum pump (Model: VE 115 N). The filtrates were then concentrated under reduced pressure (≈ 60 mbar) at 30–40 °C and 115 rpm for 6 h using a rotary evaporator (Panchun Scientific Co., Kaohsiung, Taiwan). The concentrates were frozen at − 80 °C for 24 h in an ultralow temperature freezer (Binder) before lyophilization with a freeze dryer (Alpha 1.2 LD plus) to yield a dry powder. Finally, the dried extracts were stored in dark, sealed containers at − 20 °C until further analysis.

### Plant extract analysis

The methanolic extracts of the plant species were subjected to comprehensive three-step analysis, which consisted of the evaluation of physical characteristics, preliminary qualitative phytochemical screening, and quantitative measurement of major phytochemical groups. All tests were carried out in triplicate according to standardized procedures with slight modifications.

### Physical properties of the plant extracts

Immediately after extraction, the physical properties of each methanolic extract were recorded (Table S1). Color and texture assessments were performed visually via standardized descriptive terms such as dark green, powdery, or thick, waxy, with evaluations independently verified by two researchers to reduce bias. The pH was measured in triplicate via a digital pH meter (JENWAY-3205) according to the method of [27].

### Primary qualitative phytochemical screening

The major classes of secondary metabolites in the extracts were identified following standard protocols [27]. The tested compounds included alkaloids, flavonoids, tannins, glycosides, steroids, phenols, terpenoids, saponins, carbohydrates, and proteins. These tests were detailed by [28–35]. Assays were performed in triplicate with semiquantitative scoring of reaction intensity as absent (–), low (+), moderate (++), or high (+++) [32, 34].

### Test for Alkaloids

Alkaloids were screened via Dragendorff’s test. Two milliliters of each extract were acidified with a few drops of 1% HCl and placed in three test tubes. A few drops of Dragendorff’s reagent were added to each tube. The formation of an orange‒red precipitate indicated a positive result for alkaloids.

### Test for Flavonoids

Flavonoids were detected via the ammonia/sulfuric acid test. Five mL of diluted ammonia was added to the methanolic extract, followed by 1 mL of concentrated sulfuric acid. The appearance of a yellow color indicated the presence of flavonoids.

### Test for phenols and tannins

Phenols and tannins were screened via the ferric chloride test.

- Phenols: 2 mL of plant extract was mixed with 2 mL of distilled water followed by 10% FeCl_3_ solution. A bluish-black color (which can vary to green depending on the type of phenol) indicated the presence of phenols.
- Tannins: 2 mL of 5% FeCl_3_ solution was added to 2 mL of the plant extract. The appearance of dark blue or greenish-black coloration indicated the presence of tannins.

### Test for steroids

Steroids were detected via the Liebermann–Burchard test. Two milliliters of chloroform were added to the crude extract, followed by a few drops of concentrated sulfuric acid. The formation of rose-red to reddish-brown ring in the lower chloroform layer indicated the presence of steroids.

### Test for terpenoids

Terpenoids were screened via the Salkowski test. Five milliliters of each extract were mixed with 2 mL of chloroform. One milliliter of concentrated sulfuric acid was added gently to form a layer. After mild heating for two minutes, the appearance of a reddish-brown ring at the interface indicated the presence of terpenoids.

### Test for glycosides

Glycosides were detected via the Keller–Killiani test. One milliliter extract was mixed with 1.5 mL of glacial acetic acid and one drop of 5% ferric chloride solution. Concentrated sulfuric acid was carefully added along the side of the test tube. The formation of a reddish-brown ring, which turned blue in the acetic acid layer, indicated the presence of glycosides.

### Test for saponins

Saponins were detected via the froth test, in which 2 mL of the extract was mixed with 5 mL of distilled water and shaken vigorously for 2 min, with the formation of stable and persistent foam indicating the presence of saponins.

### Test for carbohydrates

Carbohydrates were identified via Molisch’s test, where 2 mL of the extract was treated with two drops of Molisch’s reagent (α‑naphthol in ethanol), followed by the slow addition of 2 mL of concentrated sulfuric acid along the side of the test tube, and the formation of a violet ring at the interface was taken as evidence of the presence of carbohydrates.

### Testing for proteins

Proteins were detected via the Biuret test, where 2 mL of the extract was mixed with one drop of 2% copper sulfate solution, 1 mL of 95% ethanol, and a few potassium hydroxide pellets; a pink coloration in the ethanolic layer indicated the presence of proteins.

### Quantification of total phytochemical contents in the methanolic plant extracts

Quantitative analyses of total phenolic, flavonoid, and carbohydrate contents were performed using the 70% methanolic extracts of the tested plant species. The hydroalcoholic methanol–water solvent system (70:30 v/v) was chosen because it efficiently extracts both polar (*e.g*., carbohydrates, glycosides) and moderately nonpolar (*e.g*., flavonoids, phenolics) phytochemicals [36–38]. All measurements were carried out in triplicate using standardized colorimetric assays.

### Total Phenolic Content (TPC)

The total phenolic content (TPC) was estimated via the Folin–Ciocalteu spectrophotometric assay according to [39]. Briefly, 100 µL of each extract (1 mg mL^− 1^) was mixed with 150 µL of Folin–Ciocalteu reagent (containing phosphomolybdic acid and phosphotungstic acid). After allowing the mixture to stand for 5–10 min, 500 µL of 7.5% sodium carbonate (Na_2_CO_3_) solution and 400 µL of distilled water were added. The reaction mixture was then incubated in darkness at room temperature for 90 min. The absorbance was measured at 760 nm via a Thermo Scientific Evolution 100 UV–Visible spectrophotometer. The TPC was determined via a gallic acid calibration curve ranging from 50 to 500 µg mL^− 1^ with the following linear equation:

$$y=0.0074x+0.277\left(R^{2}=0.9892\right)$$

The TPC values were expressed as milligrams of gallic acid equivalents (mg GAE) per gram of dry extract.

### Total Flavonoid Contents (TFC)

The total flavonoid content (TFC) was assessed following the procedure of [40]. In this assay, 100 µL of the extract was mixed with 400 µL of 30% methanol, 100 µL of 1 M potassium acetate, and 100 µL of 0.3 M AlCl₃·6 H₂O. After allowing the mixture to stand for 5 min, 1 mL of 1 M NaOH was added, and the mixture was thoroughly mixed. The absorbance was then measured at 415 nm. A calibration curve was prepared using quercetin standards ranging from 10 to 1000 µg mL^− 1^, with the following linear equation:

$$y=0.0005x+0.0618\left(R^{2}=0.9992\right)$$

The TFC was expressed as milligrams of quercetin equivalents (mg QE) per gram of dry extract.

### Total Carbohydrate Content (TCC)

Although simple sugars are more efficiently extracted by aqueous solvents, the 70% methanolic extract used in this study contained water-soluble carbohydrate fractions, as methanol–water mixtures are known to co-extract polysaccharides, glycosides, and reducing sugars from plant tissues. Therefore, the anthrone assay was applied directly to the methanolic extract to determine TCC [41]. The absorbance was measured at 620 nm using glucose standards ranging from 10 to 100 µg mL^− 1^. The calibration curve was generated via the following linear equation:

$$y=0.0066x+0.0219\left(R^{2}=0.9989\right)$$

The TCC contents were expressed as milligrams of glucose per gram of dry extract.

### Antioxidant activity (DPPH assay)

The antioxidant potential of the extracts was determined through DPPH radical scavenging analysis, following the methodology described by [42]. A primary solution was prepared by dissolving 22 mg of DPPH in 50 mL of 99.5% ethanol and was allowed to equilibrate at 20 °C in darkness for 2 h. An operational solution was formulated by diluting the primary solution to achieve an optical density of 0.98 ± 0.02 at 517 nm. Three milliliters of the DPPH solution were mixed with 100 µL of each extract at various concentrations (25–400 µg mL⁻¹ for *D. ambrosioides*, *C. procera*, *N. oleander*, and *L. inermis*; 10–160 µg mL⁻¹ for *M. oleifera* and *A. nilotica*). The mixtures were incubated in the dark at ambient temperature for 15 min, after which the absorbance was measured at 517 nm. The radical scavenging activity (RSA) was calculated via the following formula:

$$\:\mathrm{R}\mathrm{S}\mathrm{A}\:\left(\%\right)=\frac{\mathrm{A}\mathrm{b}\mathrm{s}\mathrm{o}\mathrm{r}\mathrm{b}\mathrm{a}\mathrm{n}\mathrm{c}\mathrm{e}\:\mathrm{o}\mathrm{f}\:\mathrm{c}\mathrm{o}\mathrm{n}\mathrm{t}\mathrm{r}\mathrm{o}\mathrm{l}-\mathrm{A}\mathrm{b}\mathrm{s}\mathrm{o}\mathrm{r}\mathrm{b}\mathrm{a}\mathrm{n}\mathrm{c}\mathrm{e}\:\mathrm{o}\mathrm{f}\:\mathrm{s}\mathrm{a}\mathrm{m}\mathrm{p}\mathrm{l}\mathrm{e}}{\mathrm{A}\mathrm{b}\mathrm{s}\mathrm{o}\mathrm{r}\mathrm{b}\mathrm{a}\mathrm{n}\mathrm{c}\mathrm{e}\:\mathrm{o}\mathrm{f}\:\mathrm{c}\mathrm{o}\mathrm{n}\mathrm{t}\mathrm{r}\mathrm{o}\mathrm{l}}\:\times\:100$$

The median inhibitory concentration (IC_50_) was determined from the concentration‒response inhibition curves and expressed as the DPPH-IC_50_ at µg mL⁻¹. Ascorbic acid (50–250 µg mL⁻¹) was used as a reference standard.

### Establishment and maintenance of *Sitotroga cerealella* colonies

A breeding colony of the Angoumois grain moth, *S. cerealella*, was established using moths from naturally infested wheat obtained from the Laboratory of Insect Biology at Aswan University, Egypt. To prepare a suitable environment for the colony, healthy wheat grains were sterilized to eliminate any preexisting infestations. This process involved heat treatment, in which the grains were heat-treated at 65 ± 5 °C for 8 h. After sterilization, the grains were stored at − 20 °C for 14 days, air-dried and stored in sealed containers until use.

To establish the colony, adult moths and sterilized wheat grains were placed into 2-liter glass jars. These jars were covered with a muslin cloth and kept in larger rearing cages. To protect the colony from ants, the legs of the cages were placed in water-filled dishes. Fresh, sterilized wheat was added to the jars as needed to maintain the health of the colonies. All the moth cultures were maintained in a controlled laboratory environment with a temperature of 28 ± 2 °C and 75 ± 5% relative humidity.

### Bioassay of *Sitotroga cerealella *adults

The toxicity of the plant extracts on *S. cerealella* was assessed via sterilized wheat grains of the Sakha 95 cultivar. All adults used in the bioassay were obtained from the laboratory colony described in the previous section. Stock solutions (10% w/v; 100 mg mL^− 1^) were prepared by dissolving 10 g of dry plant material in 100 mL of methanol. From these stock solutions, serial dilutions in methanol were prepared to yield working concentrations of 1%, 3%, 5%, and 10% (corresponding to 10, 30, 50, and 100 mg mL^− 1^, respectively). Each working solution (2 mL) was thoroughly mixed with 20 g of sterilized wheat grains to achieve a uniform coating, resulting in final extract concentrations of 1, 3, 5, and 10 mg per gram of grain (equivalent to 0.1, 0.3, 0.5, and 1.0% w/w, respectively). The control grains received methanol treatment alone. Glass jars (9 × 18 cm) containing 20 g of treated wheat grains were infested with twenty newly emerged *S. cerealella* (0–24 h old). Muslin cloth covered the jars to provide adequate ventilation, while the environmental conditions were maintained at 28 ± 2 °C and 75 ± 5% relative humidity. Four replicates were established for each treatment and control. Mortality was assessed by observing the lack of responsiveness to gentle mechanical stimulus (prodding) and the absence of movement when jars were tilted. Insects exhibiting no movement were recorded as dead.

Adult mortality was recorded at 24, 48, and 72 h post-exposure. Lethal times (LT_50_ and LT_90_) were calculated for each tested concentration over a 72-hour period. The cumulative mortality of *S. cerealella* adults across all time points was expressed as the area under the curve (AUC), which was calculated via the trapezoidal rule:

$$\:AUC=\sum_{i-1}^{n-1}\frac{\left({M}_{i}+{M}_{i+1}\right)}{2}\times\:({T}_{i}-{T}_{i+1})$$

where M_i_ is the cumulative mortality (%) at time T_i_; M_i+1_ is the cumulative mortality (%) at the next time point T_i+1_; (T_i+1_ – T_i_) is the time interval between observations; and n is the total number of time points.

### Reduction in moth emergence

The impact of the extracts on *S. cerealella* adult emergence was evaluated at the same concentrations (1, 3, 5, and 10 mg g⁻¹ of grain), and the bioassay method described previously was conducted in a separate experiment.

Twenty grams of treated grains were distributed into 250 mL glass jars, with four replicates prepared for each concentration. One hundred freshly laid *S. cerealella* eggs (24 h old) were introduced into each jar. Muslin cloth was used to cover the jars to ensure adequate ventilation, and all the jars were maintained at 28 ± 2 °C and 75 ± 5% relative humidity for four weeks. Daily monitoring of adult emergence commenced after the incubation period and continued for fifteen consecutive days. Adult emergence reduction percentages were determined according to the following formula: [43]

$$\:\mathrm{R}\mathrm{e}\mathrm{d}\mathrm{u}\mathrm{c}\mathrm{t}\mathrm{i}\mathrm{o}\mathrm{n}\:\left(\%\right)=\:\frac{\mathrm{N}\mathrm{o}.\:\mathrm{o}\mathrm{f}\:\mathrm{m}\mathrm{o}\mathrm{t}\mathrm{h}\mathrm{s}\:\mathrm{e}\mathrm{m}\mathrm{e}\mathrm{r}\mathrm{g}\mathrm{i}\mathrm{n}\mathrm{g}\:\mathrm{i}\mathrm{n}\:\mathrm{t}\mathrm{h}\mathrm{e}\:\mathrm{c}\mathrm{o}\mathrm{n}\mathrm{t}\mathrm{r}\mathrm{o}\mathrm{l}-\mathrm{N}\mathrm{o}.\:\mathrm{o}\mathrm{f}\:\mathrm{m}\mathrm{o}\mathrm{t}\mathrm{h}\mathrm{s}\:\mathrm{e}\mathrm{m}\mathrm{e}\mathrm{r}\mathrm{g}\mathrm{i}\mathrm{n}\mathrm{g}\:\mathrm{i}\mathrm{n}\:\mathrm{t}\mathrm{h}\mathrm{e}\:\mathrm{t}\mathrm{r}\mathrm{e}\mathrm{a}\mathrm{t}\mathrm{m}\mathrm{e}\mathrm{n}\mathrm{t}}{\mathrm{N}\mathrm{o}.\:\mathrm{o}\mathrm{f}\:\mathrm{m}\mathrm{o}\mathrm{t}\mathrm{h}\mathrm{s}\:\mathrm{e}\mathrm{m}\mathrm{e}\mathrm{r}\mathrm{g}\mathrm{i}\mathrm{n}\mathrm{g}\:\mathrm{i}\mathrm{n}\:\mathrm{t}\mathrm{h}\mathrm{e}\:\mathrm{c}\mathrm{o}\mathrm{n}\mathrm{t}\mathrm{r}\mathrm{o}\mathrm{l}\:}\:\times\:100$$

### Protective effects on wheat grains

Following the adult emergence period of the last test, the wheat grains in each jar were sieved to remove insect-related debris. The remaining wheat from both the control and treated groups was weighed. The percentage of grain weight loss was calculated via the gravimetric (count and weight) method via the following formula:

$$\:\mathrm{W}\mathrm{e}\mathrm{i}\mathrm{g}\mathrm{h}\mathrm{t}\:\mathrm{l}\mathrm{o}\mathrm{s}\mathrm{s}\:\left(\%\right)=\:\frac{\left(\mathrm{W}\mathrm{t}.\:\mathrm{o}\mathrm{f}\:\mathrm{u}\mathrm{n}\mathrm{d}\mathrm{a}\mathrm{m}\mathrm{a}\mathrm{g}\mathrm{e}\mathrm{d}\:\mathrm{g}\mathrm{r}\mathrm{a}\mathrm{i}\mathrm{n}\mathrm{s}\times\:\mathrm{N}\mathrm{o}.\mathrm{o}\mathrm{f}\:\mathrm{d}\mathrm{a}\mathrm{m}\mathrm{a}\mathrm{g}\mathrm{e}\mathrm{d}\:\mathrm{g}\mathrm{r}\mathrm{a}\mathrm{i}\mathrm{n}\mathrm{s}\right)-(\mathrm{W}\mathrm{t}.\:\mathrm{o}\mathrm{f}\:\mathrm{d}\mathrm{a}\mathrm{m}\mathrm{a}\mathrm{g}\mathrm{e}\mathrm{d}\:\mathrm{g}\mathrm{r}\mathrm{a}\mathrm{i}\mathrm{n}\mathrm{s}\times\:\mathrm{N}\mathrm{o}.\:\mathrm{o}\mathrm{f}\:\mathrm{u}\mathrm{n}\mathrm{d}\mathrm{a}\mathrm{m}\mathrm{a}\mathrm{g}\mathrm{e}\mathrm{d}\:\mathrm{g}\mathrm{r}\mathrm{a}\mathrm{i}\mathrm{n}\mathrm{s})}{\mathrm{W}\mathrm{t}.\:\mathrm{o}\mathrm{f}\:\mathrm{u}\mathrm{n}\mathrm{d}\mathrm{a}\mathrm{m}\mathrm{a}\mathrm{g}\mathrm{e}\mathrm{d}\:\mathrm{g}\mathrm{r}\mathrm{a}\mathrm{i}\mathrm{n}\mathrm{s}\:\times\:(\mathrm{N}\mathrm{o}.\:\mathrm{o}\mathrm{f}\:\mathrm{d}\mathrm{a}\mathrm{m}\mathrm{a}\mathrm{g}\mathrm{e}\mathrm{d}\:\mathrm{g}\mathrm{r}\mathrm{a}\mathrm{i}\mathrm{n}\mathrm{s}+\mathrm{N}\mathrm{o}.\:\mathrm{o}\mathrm{f}\:\mathrm{u}\mathrm{n}\mathrm{d}\mathrm{a}\mathrm{m}\mathrm{a}\mathrm{g}\mathrm{e}\mathrm{d}\:\mathrm{g}\mathrm{r}\mathrm{a}\mathrm{i}\mathrm{n}\mathrm{s})\:}\:\times\:100$$

To calculate the IDG percentages, the number of damaged grains was divided by the total number of grains, and the results were multiplied by 100.

### Enzyme assays

#### Preparation of enzyme sources

Fourth instar larvae of *S. cerealella*, 12 h post-molt, were fed wheat grains treated with 1% (w/w) of each methanolic plant extract. To assess whether exposure to plant extracts during larval feeding causes persistent physiological alterations that carry over to the adult stage, enzyme activities were measured in newly emerged adults that had developed from treated larvae. This approach aimed to detect sublethal or residual metabolic effects resulting from larval exposure, rather than effects of direct contact in adults. Each treatment and control group consisted of three biological replicates, each comprising pooled samples of ten adults that were decapitated prior to analysis. The activities of aspartate aminotransferase (AST), alanine aminotransferase (ALT), superoxide dismutase (SOD), and reduced glutathione (GSH) were measured in whole-body homogenates, whereas alkaline phosphatase (ALP) activity was specifically assessed in midgut tissues. The colorimetric enzyme kits for these enzymes were purchased from Biodiagnostic (Dokki, Egypt).

Adults were homogenized via a Surom™ homogenizer (Model 5158 A, 50–60 Hz; Bekman Co., Egypt) in ice-cold 0.1 M phosphate buffer (pH 7.4), which was applied in three brief bursts of less than 30 s each. The homogenates were then centrifuged at 6000 rpm (≈ 4,000 × g) for 10 min at 4 °C to remove cellular debris and nuclei in a Sigma 4K15C centrifuge (Sigma Laborzentrifugen, Germany).

The midgut tissues were carefully dissected in distilled water under a stereomicroscope. The dissected midguts were immediately rinsed in the buffer before homogenization. Midgut tissues were promptly homogenized in ice-cold 0.1 M phosphate buffer (pH 10.0) and centrifuged at 10,000 rpm (≈ 11,200 × g) for 15 min at 4 °C. The resulting supernatants (crude enzyme extract) were collected as crude enzyme extracts, divided into aliquots, and stored at − 20 °C until further use. The protein contents of all the extracts were determined via the Lowry et al. method [44], which uses bovine serum albumin as the standard.

#### Alanine aminotransferase and aspartate aminotransferase

The alanine aminotransferase (ALT; EC 2.6.1.2) and aspartate aminotransferase (AST; EC 2.6.1.1) activities were assessed following the [45] method. For each assay, 0.5 mL of substrate solution (containing L-alanine (200 mM) and 2-oxoglutarate (6 mM) for ALT and L-aspartate (100 mM) and 2-oxoglutarate (5 mM) for AST) was mixed with 100 µL of homogenate and incubated at 37 °C for 30 min. Subsequently, 0.5 mL of 2,4-dinitrophenylhydrazine (12 mM) was added, followed by a 20-minute incubation at room temperature (23 ± 2 °C). Then, 5.0 mL of 0.4 M sodium hydroxide was added, and after 5 min, the absorbance was measured at 546 nm against the blank. Enzyme activities were calculated via kit conversion tables and normalized to protein content. One unit (U) was defined as the amount of enzyme required to catalyze the transformation of 1 µmol of substrate per minute under standard assay conditions. Specific activities were expressed as U mg⁻¹ protein.

#### Alkaline phosphatase

Alkaline phosphatase (ALP; EC 3.1.3.1) activity in midgut homogenates was measured via a colorimetric kit based on phenyl phosphate hydrolysis and the 4-aminophenazone reaction [46]. Sample homogenates (25 µl) were mixed with 50 µl of an alkaline buffer-substrate mixture (pH 10.0) containing phenyl phosphate (5 mM) and incubated for 20 min at 37 °C. After incubation, 250 µl of EDTA (100 mM) and 4-aminophenazone (50 mM) were added, followed by the addition of 250 µl of potassium ferricyanide (200 mM). The mixture was incubated for 5 min at room temperature in the dark, after which the absorbance was read at 510 nm via a reagent blank. A phenol standard was processed in parallel. ALP activity was calculated from the absorbance ratio, normalized to the protein concentration, and expressed as U mg⁻¹ protein.

#### Superoxide dismutase

Superoxide dismutase (SOD; EC 1.15.1.1) activity was measured via a colorimetric kit based on the nitroblue tetrazolium (NBT) reduction inhibition method [47]. The tissue homogenates (100 µL) were mixed with 1.0 mL of the working reagent containing phosphate buffer (50 mM, pH 8.5), NBT (1 mM), and NADH (1 mM). The reaction was initiated by adding 100 µL of freshly prepared phenazine methosulfate (PMS, 0.1 mM) solution, following the manufacturer’s instructions. The changes in the absorbance at 560 nm were recorded over 5 min at 25 °C. SOD activity was calculated from the percent inhibition of NBT reduction, normalized to protein content, and reported as U mg⁻¹ protein.

#### Reduced glutathione

Glutathione (GSH) was quantified via a colorimetric kit based on DTNB reduction via the method of [48]. The tissue homogenate (100 µL) was treated with 0.5 mL of trichloroacetic acid (500 mM) and centrifuged at 3000 rpm (≈ 500 x g) for 15 min at 4 °C, and the supernatant was mixed with 1.0 mL of phosphate buffer (100 mM) and 100 µL of DTNB (1.0 mM). After incubation (5 min at room temperature), the absorbance was measured at 405 nm, and the GSH content was calculated via the kit factor and normalized to the protein concentration.

#### Statistical analysis

Time–response analysis for determining LT50 and LT90 values was conducted via the LDP-line program (http://ehabsoft.com/ldpline). Prior to analysis, all the data were tested for homogeneity of variance (Levene’s test) and normality (Shapiro–Wilk test). The data were transformed when necessary to meet these assumptions. Data on phytochemical constituents, antioxidant activity (IC_50_ values) and cumulative mortality were analyzed via one-way ANOVA. Moth emergence reduction, grain weight loss, and percentages of insect-damaged grains were analyzed via two-way ANOVA to assess the effects of extract type, concentration, and their interaction. In both cases, Tukey’s HSD post hoc test was used for multiple comparisons, and differences were considered statistically significant at *P* < 0.05. Enzyme assay data were analyzed via one-way ANOVA, followed by Dunnett’s post hoc test for comparisons with the control. The percentage data for moth emergence reduction were arcsine square-root transformed prior to analysis to meet normality assumptions, whereas the table presents untransformed means ± SEs with post hoc groupings based on analyses of transformed values. All other datasets were analyzed without transformation. Linear associations among variables were assessed via Pearson’s correlation coefficient (r). Statistical analyses were conducted via SPSS software (version 22.0; IBM Corp., Armonk, NY, USA), and graphical outputs were generated via GraphPad Prism (version 9.0; GraphPad Software, San Diego, CA, USA). A significance level of *P* < 0.05 was applied for all tests.

## Results

### Primary phytochemical screening of the plant extracts

The qualitative phytochemical analysis revealed distinct variations in the distribution of secondary metabolites among the six plant extracts examined (Table S2). The plant of *D. ambrosioides* contains flavonoids, steroids, phenols, glycosides, saponins, carbohydrates, and proteins, with proteins present at moderate levels. However, alkaloids, tannins, and terpenoids were absent. The profile of the *C. procera* cultivar was similar to that of *D. ambrosioides*, with moderate amounts of steroids, saponins, and proteins. It also contains tannins at low concentrations but lacks alkaloids and terpenoids. *Moringa oleifera*, including all the tested compounds except alkaloids, presented the greatest phytochemical diversity. It is particularly rich in tannins and saponins, with moderate levels of flavonoids, steroids, and phenols. *Nerium oleander* has a broad phytochemical profile and is the only extract containing alkaloids. It has high tannin, saponin and protein contents and moderate amounts of steroids and carbohydrates, making it the extract with the highest overall secondary metabolite content. *Lawsonia inermis* presented moderate phenol content and elevated levels of saponins and carbohydrates. It lacks alkaloids, tannins, and terpenoids but contains low to moderate amounts of other metabolites. Among all the extracts, *A. nilotica* presented the highest levels of phenols, tannins, saponins, and proteins. Like most others, it does not contain alkaloids but contains moderate quantities of other secondary metabolites.

### Total content quantification of plant extracts

**Fig. 1:**
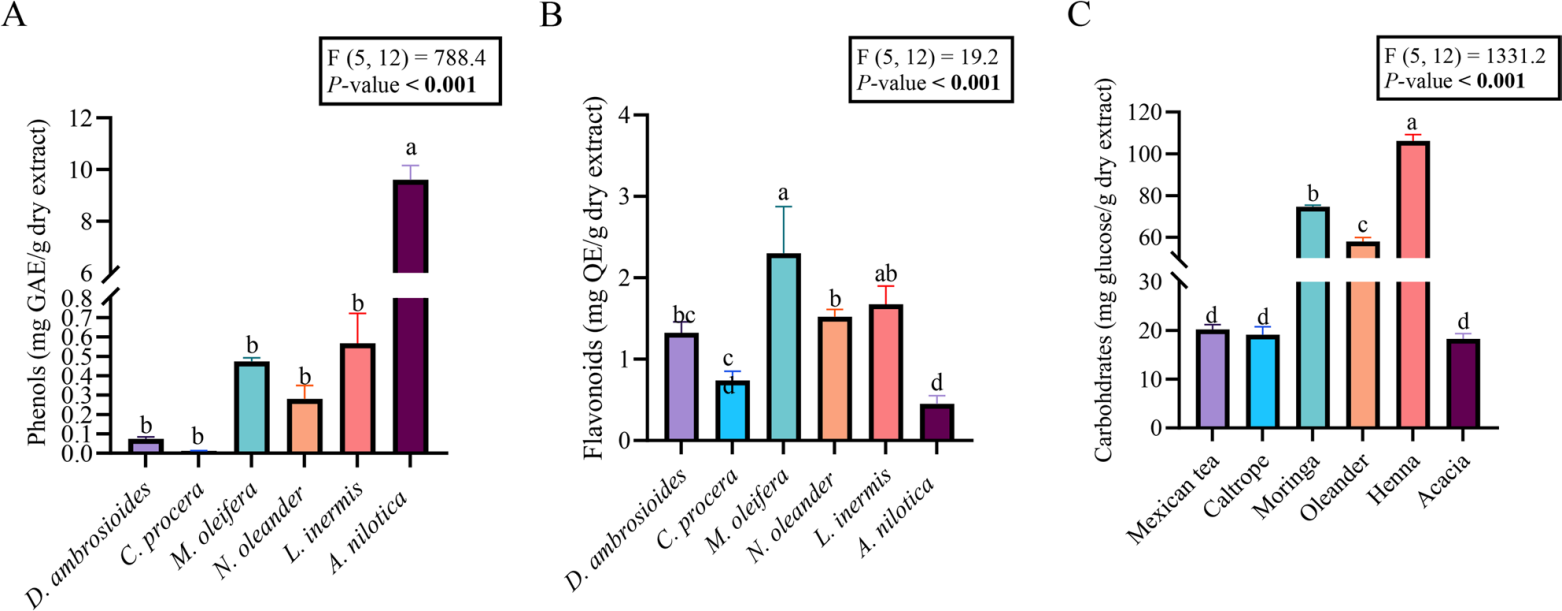
Metabolite contents of the six plant species: **A** Total phenolic content, **B** Total flavonoid content, and (**C**) Carbohydrate content. Values represent mean ± SE (*n* = 3). Different letters above the bars indicate significant differences among species assessed by Tukey’s post hoc test (*P* < 0.05)

The total phenolic content varied significantly among the six plant extracts (F_5,12_ = 788.4, *P* < 0.001). Tukey’s post hoc analysis revealed that the *A. nilotica* extract had the highest phenolic concentration, significantly exceeding those of all the other plant species assessed (Fig. 1A).

Flavonoid concentrations differed significantly across plant extracts (F_5,12_ = 19.2, *P* < 0.001). According to Tukey’s multiple comparison test, *M. oleifera* extract yielded the highest flavonoid content, significantly surpassing those of most other species. The *N. oleander* and *L. inermis* extracts presented comparable intermediate flavonoid levels that were significantly greater than those of *D. ambrosioides* and the *C. procera* but lower than those of *M. oleifera*. The *A. nilotica* extracts presented relatively low flavonoid contents (Fig. 1B).

Carbohydrate concentrations were significantly influenced by plant species (F_5,12_ = 1331.2, *P* < 0.001). Tukey’s test indicated that the *L. inermis* extract presented the highest carbohydrate content, which was significantly different from that of all the other treatments. *Moringa oleifera* extract presented the second-highest carbohydrate level, followed by *N. oleander* extract, both of which presented intermediate concentrations. The *D. ambrosioides*, *C. procera*, and *A. nilotica* extracts presented similar and relatively lower carbohydrate contents (Fig. 1C).

### Antioxidant potential (DPPH-IC_50_)

The linear regression equations, coefficient of determination (R^2^), and IC_50_ values (µg mL^− 1^) for the DPPH radical scavenging activity of the tested plant extracts are shown in Table 2. The antioxidant potential, as indicated by the DPPH-IC_50_ values, varied significantly among the six plant extracts (F_5,12_ = 224.0, *P* < 0.001). According to Tukey’s multiple comparison test, *A. nilotica* (59.43 µg mL^− 1^) had the lowest IC_50_ value of all the samples, reflecting the strongest antioxidant potential, and was significantly different from all the other extracts. *Lawsonia inermis* (236.1 µg mL^− 1^) presented the highest IC_50_ value, which was significantly greater than those of all the other extracts, indicating that *L. inermis* had the lowest antioxidant activity. *Dysphania ambrosioides* (172.6 µg mL^− 1^) presented a moderately high IC_50_ value, which was statistically distinct from that of *L. inermis* and most other species. *Calotropis procera* (102.1 µg mL^− 1^), *M. oleifera* (87.03 µg mL^− 1^), and *N. oleander* (101.2 µg mL^− 1^) were statistically similar to each other, with intermediate IC_50_ values.

**Table 2:** Linear regression equations, coefficient of determination (R2), and IC_50_ values (µg mL^− 1^) for the DPPH radical scavenging activity of the tested plant extracts

Extract	Equation	*R*^2^	IC_50_ (µg mL^− 1^)
*Dysphania ambrosioides*	y = 0.1489x + 15.97	0.96	172.6 ± 3.23 ^b^
*Calotropis procera*	y = 0.3112x + 12.24	0.92	102.1 ± 1.63 ^c^
*Moringa oleifera*	y = 0.4005x + 8.417	0.96	87.03 ± 12.5 ^c^
*Nerium oleander*	y = 0.3085x + 12.49	0.91	101.2 ± 1.77 ^c^
*Lawsonia inermis*	y = 0.1295x + 13.66	0.92	236.1 ± 7.23 ^a^
*Acacia nilotica*	y = 0.4455x + 12.56	0.96	59.43 ± 2.14 ^d^
F_(5, 12)_			224.0
*P*			< 0.001

The values represent the means ± SEs. Different letters indicate significant differences among species according to Tukey’s post hoc test (*P* < 0.05)

The values represent the means ± SEs. Different letters indicate significant differences among species according to Tukey’s post hoc test (*P* < 0.05).

### Toxicity assay on *Sitotroga cerealella* adults

**Fig. 2:**
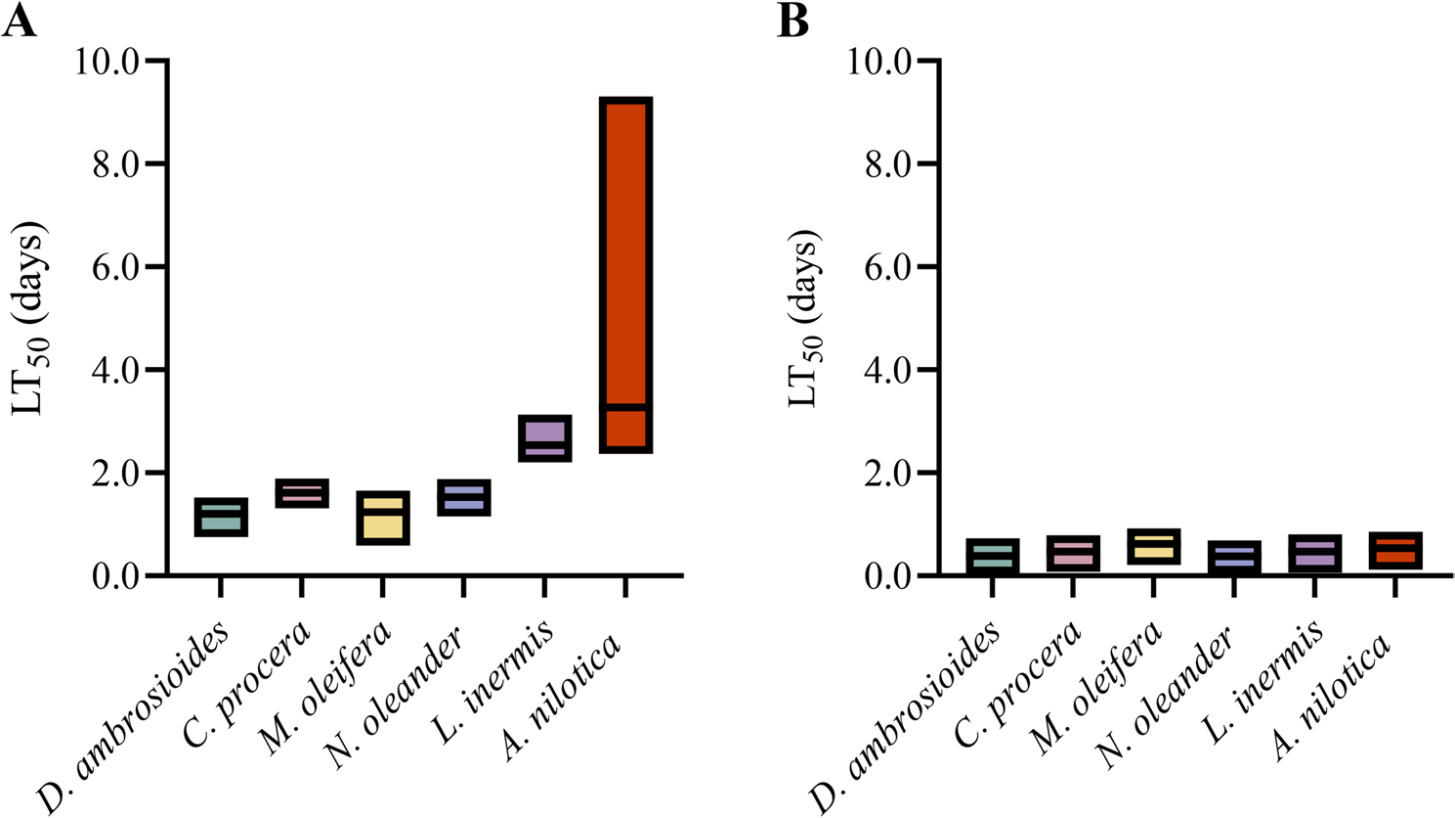
Summary of LT_50_ values and their confidence limits for *Dysphania ambrosioides*, *Calotropis procera*, *Moringa oleifera*, *Lawsonia inermis*, and *Acacia nilotica*, at (**A**) 1 mg g^− 1^, and (**B**) 10 mg g^− 1^ against *Sitotroga cerealella* adults. Full LT values and slope parameters are provided in Table S3

The lethal time (LT_50_ and LT_90_) values with 95% confidence limits and slope values of the six tested plant extracts were determined at concentrations of 1, 3, 5, and 10 mg g^− 1^ (Table S3). The results indicate variation in toxicity dynamics among extracts and concentrations, with the LT_50_ generally decreasing at higher doses. At 1 mg g^− 1^, *D. ambrosioides* presented the lowest LT_50_ value, followed by *M. oleifera*, *N. oleander*, *C. procera*, *L. inermis*, and finally *A. nilotica*, with LT_50_ values of 1.20, 1.24, 1.53, 1.61, 2.54, and 3.27 days, respectively (Fig. 2A). At 3 mg g^− 1^, the order was *D. ambrosioides*, *M. oleifera*, *A. nilotica*, *N. oleander*, *C. procera*, and finally *L. inermis*, with LT_50_ values of 0.68, 0.76, 1.03, 1.08, 1.18, and 2.06 days, respectively. At 5 mg g^− 1^, the sequences were *N. oleander*, *D. ambrosioides*, *C. procera*, *M. oleifera*, *A. nilotica*, and *L. inermis*, with LT_50_ values of 0.50, 0.56, 0.71, 0.72, 0.84, and 1.04 days, respectively. At 10 mg g^− 1^, the order was *N. oleander*, *D. ambrosioides*, *C. procera*, *L. inermis*, *A. nilotica*, and *M. oleifera*, with LT_50_ values of 0.38, 0.39, 0.47, 0.47, 0.54, and 0.62 days, respectively (Fig. 2B).

### Cumulative mortality

**Fig. 3:**
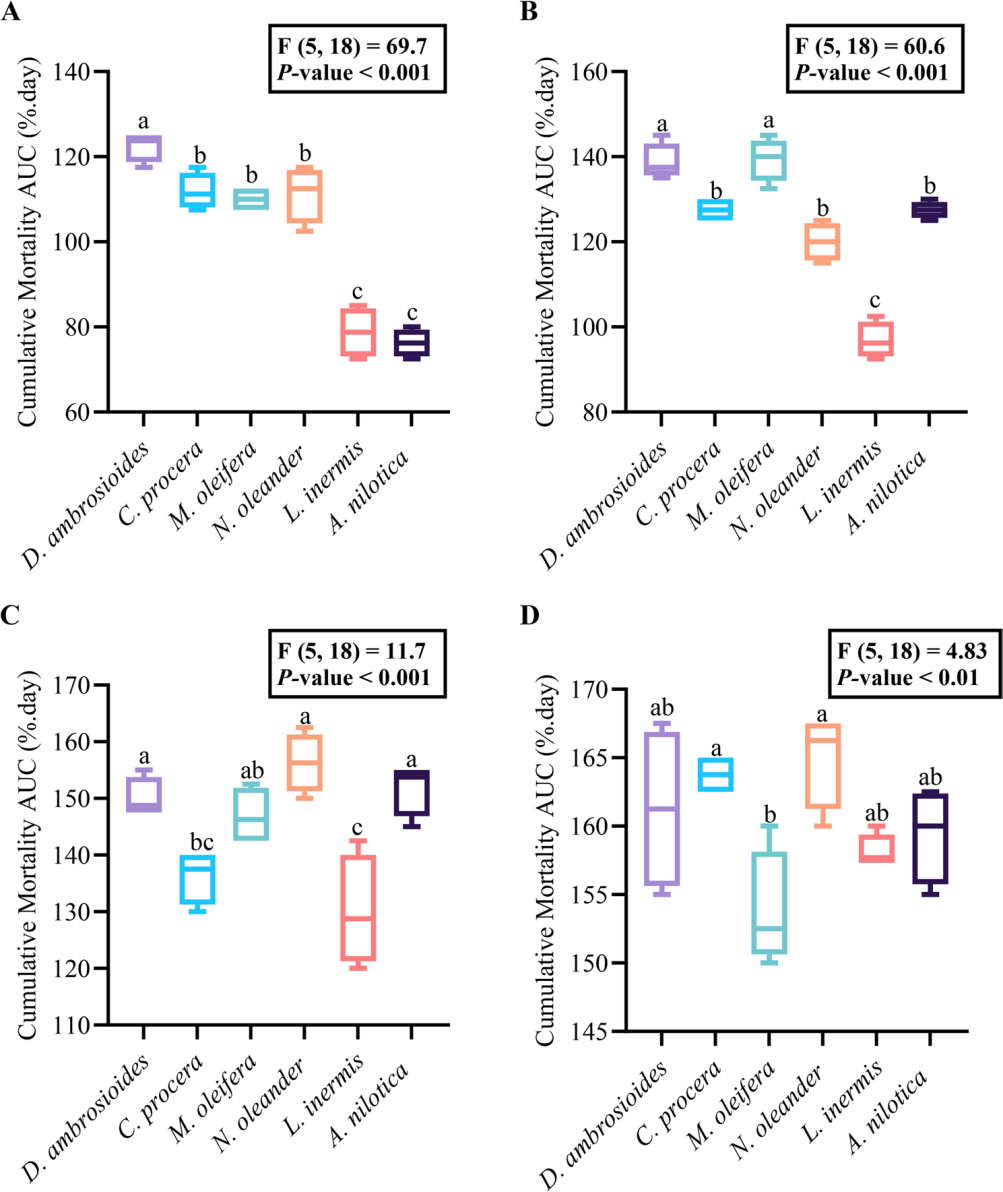
Cumulative mortality expressed as AUC (%.day) of *Sitotroga cerealella* adults over three days of exposure to *Dysphania ambrosioides*, *Calotropis procera*, *Moringa oleifera*, *Lawsonia inermis*, and *Acacia nilotica*, at (**A**) 1 mg g^− 1^, (**B**) 3 mg g^− 1^, (**C**) 5 mg g^− 1^, and **D**)10 mg g^− 1^. Boxplots represent minimum and maximum values per extract (*n* = 4). Different letters above boxes indicate significant differences among extracts within each concentration according to Tukey’s HSD test (*P* < 0.05). Cumulative mortality (AUC) calculated using the trapezoidal-rule formula. Control mortality remained 0%, yielding AUC = 0%·day (not shown)

The cumulative mortality of *S. cerealella* adults, expressed as the AUC, was significantly affected by the plant extract and concentration (Fig. 3A-D). At 1 mg g^− 1^, *D. ambrosioides* caused the greatest mortality, whereas *L. inermis* and *A. nilotica* had the weakest effects. *Calotropis procera*, *N. oleander*, and *M. oleifera* produced intermediate responses (Fig. 3A). At 3 mg g^− 1^, mortality remained highest for *D. ambrosioides* and *M. oleifera*, moderate for *C. procera*,* N. oleander* and *A. nilotica*, and lowest for *L. inermis* (Fig. 3B). At 5 mg g^− 1^, *D. ambrosioides*, *N. oleander*, and *A. nilotica* showed superior performance, while *C. procera* and *M. oleifera* produced intermediate values, and *L. inermis* was the least effective (Fig. 3C). At the maximum concentration (10 mg g^− 1^), the differences among the extracts diminished, although *N. oleander* and *C. procera* retained the highest activity, *M. oleifera* presented the lowest activity, and the remaining extracts did not differ significantly (Fig. 3D).

Overall, mortality was consistently greater with *D. ambrosioides* and *N. oleander*, whereas *L. inermis* and *M. oleifera* tended to be least effective, with *C. procera* and *A. nilotica* producing variable but intermediate effects. The solvent control showed 0% mortality at all observation times, resulting in an AUC = 0%.day; therefore, it was not plotted in the cumulative-mortality figure.

### Adult emergence reduction

Adult emergence reduction was significantly influenced by extract type (F_5,72_ = 60.03, *P* < 0.001), extract concentration (F_3,72_ = 1003.25, *P* < 0.001), and their interaction (F_15,72_ = 5.38, *P* < 0.001). The emergence reduction increased steadily with concentration, ranging from 61.5% at 1 mg g^− 1^ to 94.96% at 10 mg g^− 1^ (Table 3 and Table S4 ).

Across all the concentrations, *D. ambrosioides* consistently produced the greatest reduction (86.73%), followed by *A. nilotica* (82.34%), whereas *L. inermis* was the least effective (74.51%), which was significantly lower than that of all the other extracts (*P* < 0.05). *Calotropis procera* (79.64%) and *M. oleifera* (79.17%) had intermediate activities, which were statistically similar to each other, whereas *N. oleander* (77.77%) was slightly less effective (Table 3).

At the lowest concentration (1 mg g^− 1^), *L. inermis* reduced adult emergence by only 52.92%, which was significantly lower than that of all other extracts, while *D. ambrosioides* reached 71.58%. At 3 mg g⁻¹, the reduction ranged from 70.50% (*L. inermis*) to 83.93% (*D. ambrosioides*). At 5 mg g⁻¹, all the extracts achieved a ≥ 79% reduction, with *D. ambrosioides* again having the greatest reduction (93.15%) and *L. inermis* having the lowest reduction (79.72%). At 10 mg g^− 1^, all extracts exceeded 90%, with *D. ambrosioides* (98.26%), *A. nilotica* (97.14%), and *M. oleifera* (96.39%) showing the strongest effects (Table 3). Overall, emergence reduction decreased in the following order: *D. ambrosioides* ≈ *A. nilotica* > *M. oleifera* ≈ *C. procera* > *N. oleander* > *L. inermis*. Control emergence served as the baseline for all reduction calculations.

**Table 3:** Adult emergence reduction (%) of *Sitotroga Cerealella* exposed to plant extracts at different concentrations

Extract	1 mg g⁻¹ (% ± SE)	3 mg g⁻¹ (% ± SE)	5 mg g⁻¹ (% ± SE)	10 mg g⁻¹ (% ± SE)	Total (% ± SE)
*Dysphania ambrosioides*	71.58 ± 0.86 ᵃ	83.93 ± 1.16 ᵃ	93.15 ± 0.37 ᵃ	98.26 ± 0.43 ᵃ	86.73 ± 2.64 ^A^
*Calotropis procera*	61.88 ± 1.56 ᵇᶜ	77.21 ± 0.77 ᵇᶜ	87.18 ± 0.71 ᵃᵇ	92.29 ± 0.43 ᵇ	79.64 ± 3.03 ^BC^
*Moringa oleifera*	57.77 ± 1.28 ᶜ	74.60 ± 1.79 ᶜ	87.92 ± 0.37 ᵃᵇ	96.39 ± 0.87 ᵃ	79.17 ± 3.81 ^BC^
*Nerium oleander*	61.13 ± 1.83 ᵇᶜ	74.60 ± 0.84 ᶜ	84.57 ± 0.61 ᵇ	90.80 ± 0.83 ᵇ	77.77 ± 2.94 ^C^
*Lawsonia inermis*	52.92 ± 0.96 ᵈ	70.50 ± 1.04 ᵈ	79.72 ± 1.27 ᶜ	94.90 ± 1.06 ᵃᵇ	74.51 ± 3.96 ^D^
*Acacia nilotica*	63.74 ± 1.42 ᵇ	81.32 ± 0.52 ᵃᵇ	87.18 ± 0.37 ᵃᵇ	97.14 ± 0.72 ᵃ	82.34 ± 3.16 ^B^
Total	61.50 ± 1.28 ^d^	77.03 ± 1.02 ^c^	86.62 ± 0.87 ^b^	94.96 ± 0.62 ^a^	80.03 ± 1.36

Values are untransformed means ± SE of % adult emergence reduction. Data were analyzed after arcsine square-root transformation to meet normality assumptions (Tukey’s HSD, *P* < 0.05). Superscript lowercase letters within each column indicate significant differences among extracts at the same concentration. Superscript uppercase letters in the ‘Total’ column indicate differences among extracts averaged across concentrations. Superscripts in the ‘Total’ row indicate significant differences among concentrations

### Grain protection efficacy

Grain weight loss (%) was significantly influenced by extract type (F_5,75_ = 8.86, *P* < 0.001), extract concentration (F_3,75_ = 72.59, *P* < 0.001), and their interaction (F_15,75_ = 3.47, *P* < 0.001) (Table S5).

**Fig. 4:**
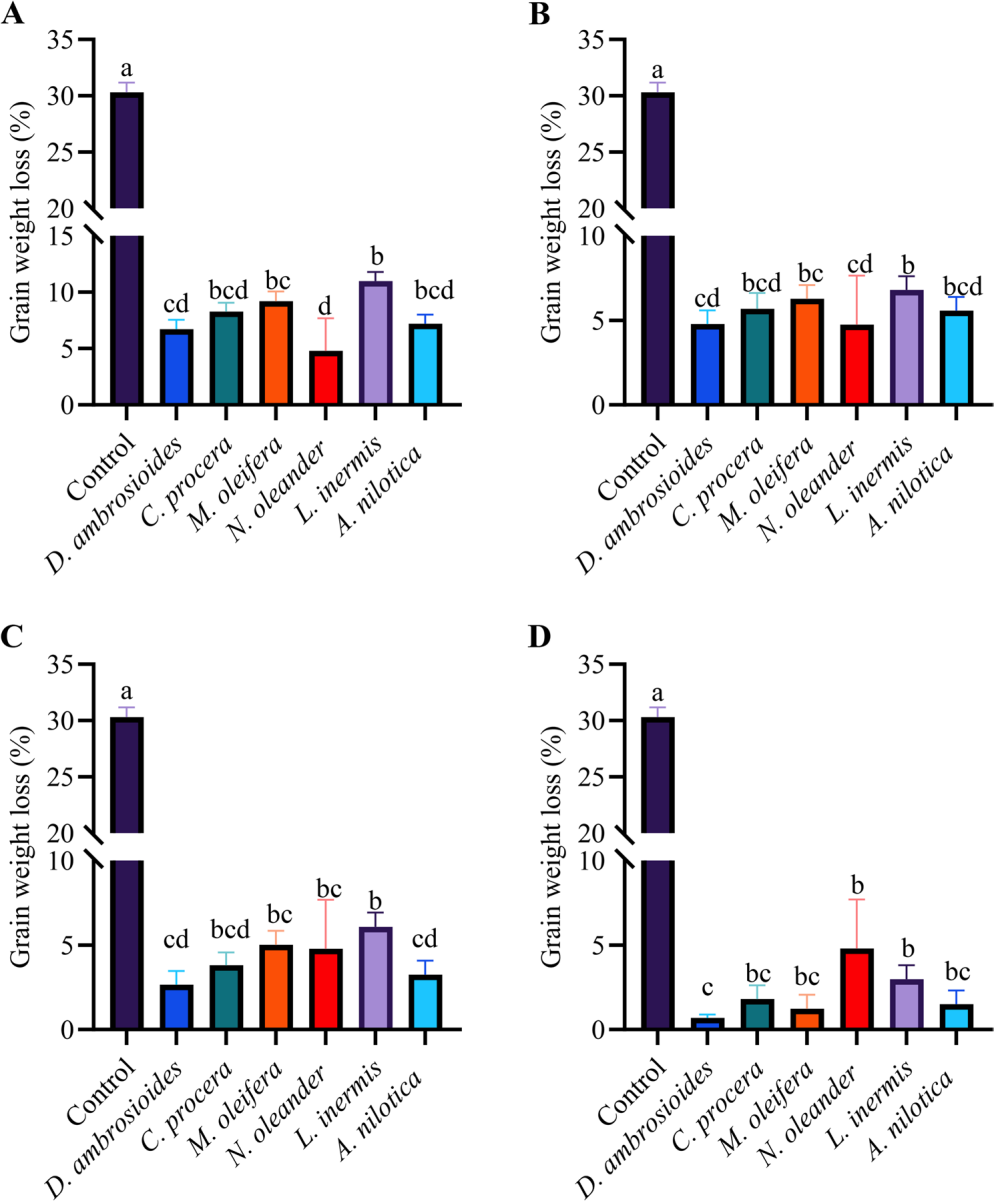
Impact of six plant extracts on grain weight loss (%) at four concentrations. Panels show mean ± SE at (**A**) 1 mg g^− 1^, (**B**) 3 mg g^− 1^, (**C**) 5 mg g^− 1^, and (**D**) 10 mg g^− 1^ (*n* = 4). Different letters above bars indicate significant differences among extracts within each concentration according to Tukey’s HSD test (*P* < 0.05) performed following a two-way ANOVA. Comparisons were conducted within each concentration level when a significant interaction was detected

Across all the concentrations, the control group presented substantially greater weight loss (30.31%) than did all the treatment groups (Fig. 4). Compared with the control, all the extracts significantly reduced weight loss (*P* < 0.001). Among the extracts, *D. ambrosioides*, *A. nilotica*, *C. procera*, and *N. oleander* consistently produced the lowest weight loss values, indicating strong protective effects. At 10 mg g^− 1^, *D. ambrosioides* was the most effective, reducing weight loss to 0.69%, followed by *A. nilotica* (1.51%) and *C. procera* (1.81%) (Fig. 4D). In contrast, *L. inermis* exhibited the weakest protection at 1 mg g^− 1^ (10.99%), followed by *M. oleifera*(9.05%) and *C. procera* (8.00%) (Fig. 4A). Although *L. inermis* and *M. oleifera* reduce weight loss, they are generally less effective than other extracts are, resulting in greater residual losses at all concentrations (e.g., *L. inermis* = 2.98% at 10 mg g⁻¹). Increasing the concentration from 1 mg g^− 1^ to 10 mg g^− 1^ significantly reduced weight loss across all the extracts (*P* < 0.001), confirming a dose-dependent protective effect (Table S5).

#### Insect-damaged grains

**Fig. 5:**
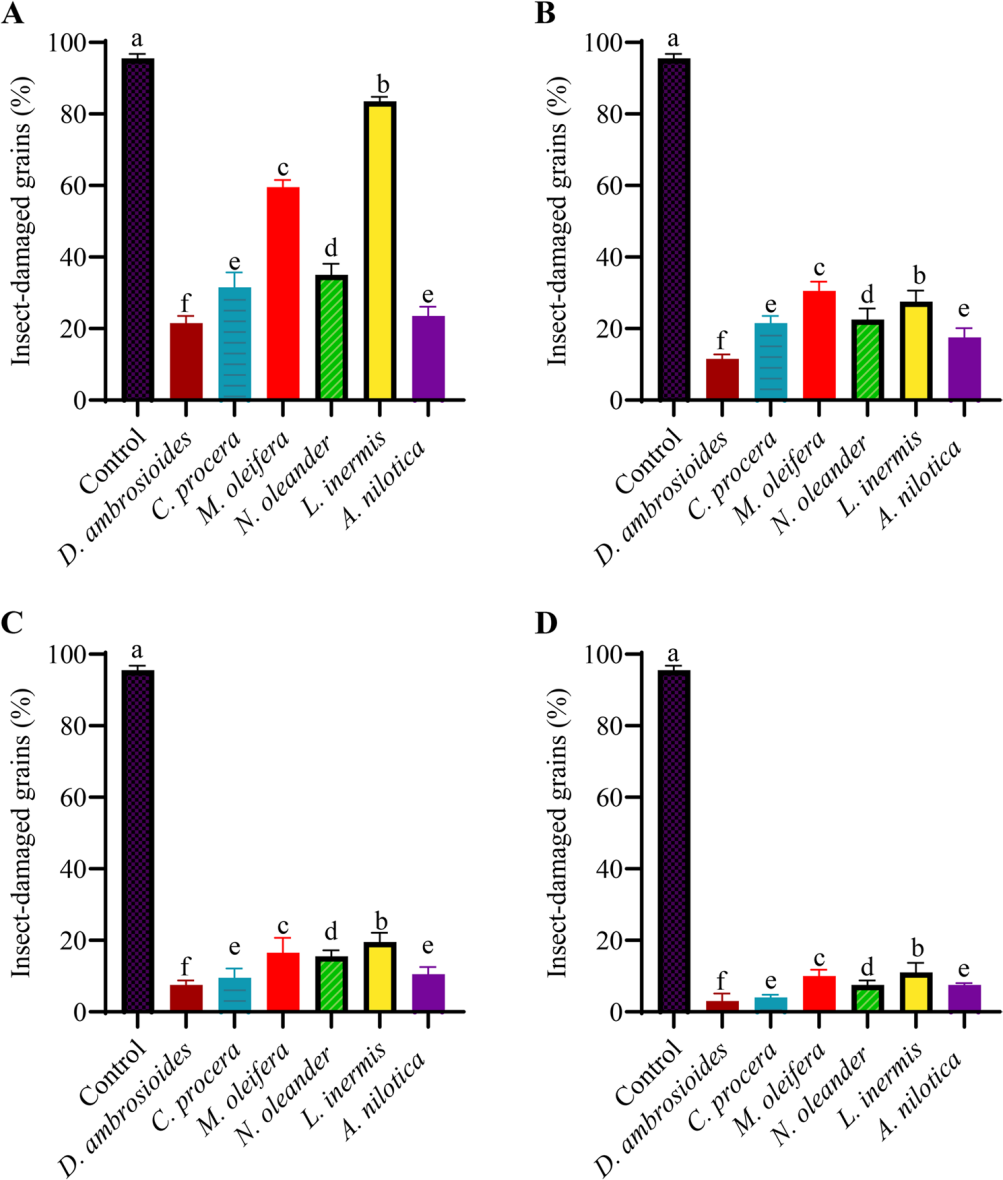
Insect-damaged grains (%) after treatment with six different plant extracts—*Dysphania ambrosioides*, *Calotropis procera*, *Moringa oleifera*, *Nerium oleander*, *Lawsonia inermis*, and *Acacia nilotica*—applied at four concentrations (1, 3, 5, and 10 mg g⁻¹, labeled **A**, **B**, **C**, and **D**, respectively) (*n* = 4). Different letters above bars indicate significant differences among extracts within each concentration according to Tukey’s HSD test (*P* < 0.05) performed following a two-way ANOVA. Comparisons were conducted within each concentration level when a significant interaction was detected

Insect damage was significantly affected by extract type (F_5,75_ = 240.01, *P* < 0.001), concentration (F_3,75_ = 984.34, *P* < 0.001), and their interaction (F_15,75_ = 74.02, *P* < 0.001) (Table S6). The control group presented the greatest degree of grain damage (95.5%), which was significantly greater than that in all the treatment groups (*P* < 0.001) (Fig. 5).

Among the extracts, *D. ambrosioides* consistently exhibited the strongest protective effect, resulting in the lowest degree of insect damage across all concentrations (21.5% at 1 mg g⁻¹ and 3.00% at 10 mg g⁻¹). *Acacia nilotica* (23.5% at mg g^− 1^ and 7.5% at 10 mg g^− 1^) and *C. procera* (31.5% at 1 mg g⁻¹ and 4.00% at 10 mg g⁻¹) ranked next in efficacy, maintaining significantly lower damage levels than *N. oleander* (35.0% at 1 mg g⁻¹ and 7.50% at 10 mg g⁻¹), *M. oleifera* (59.5% at 1 mg g⁻¹ and 10.0% at 10 mg g⁻¹), and *L. inermis* (83.5% at 1 mg g⁻¹ and 11.0% at 10 mg g⁻¹) (Fig. 5A-D).

#### Enzyme assays

**Fig. 6:**
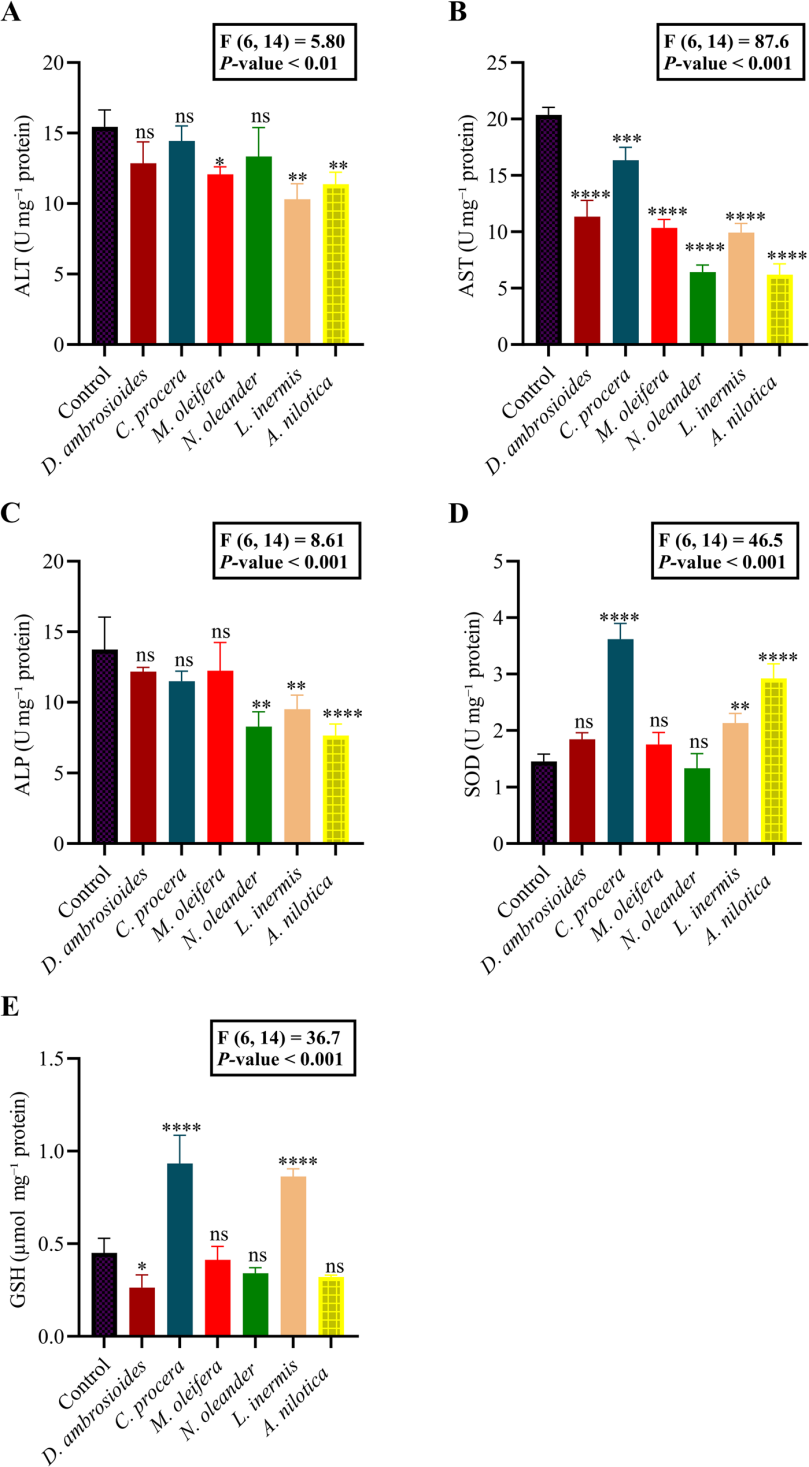
Impacts of plant extracts (*Dysphania ambrosioides*, *Calotropis procera*, *Moringa oleifera*, *Nerium oleander*, *Lawsonia inermis*, and *Acacia nilotica* ) on (**A**) ALT, (**B**) AST, (**C**) ALP, (**D**) SOD, and (**E**) GSH levels in *Sitotroga cerealella*. Data represents ± SE (*n* = 3). Statistical analysis was conducted using one-way ANOVA followed by Dunnett’s post hoc test, comparing each treatment group to the control. *P* < 0.05 considered statistically significant. Significance indicators (relative to the control): ns, not significant; *P < 0.05; **P < 0.01; ***P < 0.001; ****P < 0.0001

One-way ANOVA revealed significant effects of plant extract treatments on all measured parameters, except where indicated (Fig. 6A-E).

For ALT activity, there was a significant overall effect, F_6,14_ = 5.80, *P* = < 0.01. Dunnett’s post hoc test revealed that *M. oleifera* (*P* < 0.05), *L. inermis* (*P* < 0.01), and *A. nilotica* (*P* < 0.01) significantly reduced ALT levels compared with the control, whereas *D. ambrosioides*, *C. procera*, and *N. oleander* did not significantly differ (*P* > 0.05) (Fig. 6A).

For AST activity, treatment effects were highly significant, F_6,14_ = 87.58, *P* < 0.001. All extracts (*D. ambrosioides*, *C. procera*, *M. oleifera*, *N. oleander*, *L. inermis*, and *A. nilotica*) significantly reduced AST levels compared to the control (*P* < 0.0001 for all) (Fig. 6B).

For ALP activity, ANOVA indicated a significant effect of treatment, F_6,14_ = 8.61, *P* < 0.001. *Lawsonia inermis* (*P* < 0.01), *A. nilotica* (*P* < 0.0001) and *N. oleander* (*P* < 0.01) significantly lowered ALP levels, while *D. ambrosioides*, *C. procera*, and *M. oleifera* showed no significant differences from control (Fig. 6C).

For SOD activity, there was a strong treatment effect, F_6,14_ = 46.54, *P* < 0.001. *Calotropis procera* (*P* < 0.0001), *L. inermis* (*P* < 0.01), and *A. nilotica* (*P* < 0.0001) significantly increased SOD activity, whereas *D. ambrosioides*, *M. oleifera*, and *N. oleander* did not differ from control (Fig. 6D).

For GSH levels, ANOVA showed a significant treatment effect, F_6,14_ = 36.70, *P* < 0.001. *Calotropis procera* (*P* < 0.0001), *L. inermis* (*P* < 0.0001), and *D. ambrosioides* (*P* < 0.05) significantly increased GSH compared to the control, while *M. oleifera*, *N. oleander*, and *A. nilotica* showed no significant differences (*P* > 0.05) (Fig. 6E).

### Correlations between phytochemical constituents, antioxidant potential, and insect biological/grain protection parameters

The Pearson correlation analysis revealed that phenols had a significant negative correlation with the DPPH-IC_50_ (*r* = − 0.480, *P* < 0.05), indicating that a higher phenolic content is associated with greater antioxidant activity (Table 4). No significant correlations were detected between phenols and ER, WL, or IDG. Flavonoids demonstrated a strong positive and highly significant correlation with IDG (*r* = 0.600, *P* < 0.01), but their correlations with DPPH IC_50_, ER, and WL were not statistically significant. Carbohydrates were significantly and positively correlated with DPPH-IC_50_ (*r* = 0.541, *P* < 0.05), WL (*r* = 0.612, *P* < 0.01), and IDG (*r* = 0.941, *P* < 0.01) but strongly negatively correlated with ER (*r* = − 0.828, *P* < 0.01). Finally, the DPPH-IC_50_ was positively correlated with IDG (*r* = 0.586, *P* < 0.05), suggesting that lower antioxidant activity is associated with greater insect development/grain damage.

**Table 4:** Pearson correlation coefficients (r) between phytochemical constituents, antioxidant potential (DPPH-IC_50_), and insect biological/grain protection parameters

Variable	DPPH-IC_50_ (µg mL^− 1^)	ER (%)	WL (%)	IDG
Phenols	-0.480*	0.156	-0.157	-0.338
Flavonoids	0.284	-0.450	0.337	0.600**
Carbohydrates	0.541*	-0.828**	0.612**	0.941**
DPPH-IC_50_	1.000	-0.269	0.463	0.586*

*ER* Emergence Reduction, *WL* Grain Weight Loss, *IDG* Insect-Damaged Grains

*Significant at *P* < 0.05; **Significant at *P* < 0.01

### Correlations between phytochemical constituents, antioxidant potential, and enzyme activities

Pearson correlation analysis revealed that phenols were significantly negatively correlated with AST (*r* = − 0.528, *P* < 0.05) and ALP (*r* = − 0.579, *P* < 0.05) but not significantly correlated with ALT, SOD, or GSH (Table 5). Flavonoids exhibited a strong and highly significant negative correlation with SOD (*r* = − 0.696, *P* < 0.01), but their correlations with other enzymes were not significant. Carbohydrates were significantly and negatively correlated with ALT (*r* = − 0.478, *P* < 0.05) and SOD (*r* = − 0.490, *P* < 0.05) but not with AST, ALP, or GSH. DPPH-*IC50* was not significantly correlated with any of the measured enzyme activities.

**Table 5:** Pearson correlation coefficients (r) between phytochemical constituents, antioxidant potential (DPPH-IC_50_), and insect enzyme activities

Variable	ALT	AST	ALP	SOD	GSH
Phenols	-0.307	-0.528*	-0.579*	0.339	-0.322
Flavonoids	-0.177	-0.064	0.264	-0.696**	-0.049
Carbohydrates	-0.478*	-0.201	-0.060	-0.490*	0.296
DPPH-IC_50_	-0.316	0.174	0.158	-0.245	0.376

*ALT* Alanine Aminotransferase, *AST* Aspartate aminotransferase, *ALP* Alkaline phosphatase, *SOD* Superoxide Dismutase, *GSH* Reduced Glutathione

*Significant at *P* < 0.05; **Significant at *P* < 0.01

## Discussion

The six botanical extracts exhibited distinct phytochemical signatures that were translated into clearly differentiated antioxidant and insecticidal responses against *S. cerealella*. These contrasts underline that activity arises from dominant compound classes rather than total metabolite abundance.

### Phytochemical composition and antioxidant activity

*Acacia nilotica*, the richest in phenols and tannins, displayed the highest antioxidant capacity (lowest DPPH-IC_50_ = 59.43 µg mL^− 1^). The significant negative correlation between total phenolics and DPPH-IC_50_ (*r* = − 0.480) confirms phenolics as primary free-radical quenchers, consistent with reports that hydroxylated polyphenols are the main determinants of antioxidant power in plant extracts [25, 49]. In contrast, *L. inermis*, dominated by carbohydrates and poor in phenols, had the weakest antioxidant activity (IC_50_ = 236 µg mL^− 1^) and contributed to the observed positive correlation between carbohydrates and IC_50_ (*r* = 0.541). This apparent paradox is biologically plausible. In an insect, extracts can act as pro-oxidant stressors via (i) redox-cycling quinones that generate ROS [49]; (ii) mitochondrial interference that elevates superoxide; (iii) membrane damage by saponins provoking oxidative/immune responses [50]. Thus, SOD/GSH reflects oxidative burden, not merely chemical quenching capacity and are best interpreted as stress biomarkers rather than surrogates for DPPH.

*Moringa oleifera* was distinguished among the screened botanicals by possessing the highest qualitative and quantitative flavonoid content, alongside substantial levels of phenols and tannins, as confirmed by both phytochemical tests and total content assays. While this biochemical diversity suggested a strong antioxidant profile, the measured antioxidant efficiency was only moderate (DPPH IC_50_ ≈ 87 µg mL^− 1^), highlighting that high flavonoid concentrations alone do not guarantee maximal antioxidant capacity in this species. This observation is consistent with several independent studies examining *M. oleifera* leaf extracts, which likewise report robust flavonoid and phenolic content but only moderate-to-strong antioxidant activity—attributable to synergistic effects among phenolics, tannins, and other metabolites, rather than flavonoids alone [51–53]. Conversely, such a disconnect has not been found universally; in *M. oleifera* and other species like *Ocimum tenuiflorum*, high flavonoid content does track closely with increased antioxidant power [54]. These species differences reinforce that the predictive value of flavonoid levels is context-dependent and linked to the extract matrix and the profile of accompanying phytochemicals.​​.

### Contact toxicity and rapid mortality

These compositional differences were mirrored in the toxic outcomes. *Dysphania ambrosioides* and *N. oleander* produced the lowest LT_50_ values and highest cumulative mortality, confirming their rapid-acting nature. This aligns with prior studies identifying *D. ambrosioides* as a powerful insecticide against several pests [55–59]. Notably, the insecticidal strength of *D. ambrosioides* is frequently attributed to its monoterpene-rich essential oils (e.g., ascaridole, p-cymene, and terpinolene), which are known for their potent insect toxicity and fumigant action [60]. Furthermore, *N. oleander’s* strong mortality and grain-loss reduction reflect the presence of alkaloids and cardiac glycosides known to interfere with ion transport and neural transmission [11, 25]. However, its broad vertebrate toxicity [11, 61] limits its direct field use and suggests its value lies in isolating safer derivatives or employing controlled formulations.

### Long-term protection and sublethal effects

Slower but cumulative effects characterized *A. nilotica*. It reduced adult emergence and grain damage in a concentration-dependent manner. In *A. nilotica*, phenolic- and tannin-driven digestive inhibition explains its strong protection despite modest acute mortality. The significant negative correlations between phenols and AST (*r* = − 0.53) and ALP (*r* = − 0.58) indicate suppression of metabolic and gut enzymes, in line with tannin-mediated enzyme binding and nutrient deprivation [62, 63].

*Calotropis procera* combined intermediate mortality with significant SOD and GSH induction, denoting oxidative stress triggered by membrane-active saponins [50]. These effects match earlier descriptions of saponin-induced ROS generation and detoxifying-enzyme activation in stored-product pests [64]. *Lawsonia inermis* presented an opposite pattern: low in-vitro antioxidant capacity but strong in-vivo oxidative responses, explained by redox cycling of lawsone (2-hydroxy-1,4-naphthoquinone) that produces reactive oxygen species [49]. Thus, its elevated SOD and GSH reflect pro-oxidant stress rather than antioxidant protection, illustrating that chemical radical-scavenging ability does not necessarily predict biological oxidative effects.

In the current study, although no synthetic insecticide or fumigant was tested in parallel, the antioxidant and insecticidal activities observed in the tested plant extracts were consistent with the oxidative effects reported for several conventional insecticides and fumigants, supporting their potential as safer botanical alternatives for stored-grain pest management [65–70]. Moreover, compared with earlier reports on plant-based protectants, these findings extend existing models by demonstrating that antioxidant potential, enzyme modulation, and toxicity form a coherent continuum rather than independent effects.

Because enzyme activities were assessed in newly emerged adults that had developed from treated larvae, the observed reductions in AST, ALT, and ALP represent developmental carry-over rather than direct adult exposure. Larval ingestion of phytochemicals appears to leave persistent metabolic imprints detectable in adults, explaining continued suppression of fitness even after contact ceases. Similar trans-stadial enzyme disruptions have been documented for the same insect species and other stored product insects exposed to botanical toxins during immature stages [7, 71]. This developmental perspective provides a mechanistic rationale for long-lasting grain protection in the absence of residual contact toxicity.

Correlation analysis reinforces these mechanistic interpretations. Phenolic content correlated inversely with AST and ALP, confirming the digestive-enzyme inhibition route. Flavonoid and carbohydrate contents correlated negatively with SOD (*r* = − 0.696 and − 0.490), suggesting that antioxidant-rich or sugar-rich extracts quench reactive oxygen species and reduce enzymatic induction. Conversely, saponin- and quinone-rich extracts (*C. procera*, *L. inermis*) markedly elevated SOD and GSH, consistent with oxidative-stress induction. Collectively, these patterns reveal three interacting biochemical pathways: (i) phenolic/tannin-mediated digestive inhibition and metabolic depression; (ii) saponin- or quinone-induced oxidative stress; and (iii) volatile or glycoside-driven neurotoxicity.

Two contributions are especially noteworthy: (1) this is among the first demonstrations of persistent larva-to-adult metabolic impairment in *S. cerealella* following larval exposure to botanicals; and (2) it provides the first mechanistic explanation for *L. inermis* activity against this pest, rooted in redox-cycling naphthoquinones. Together, the results establish a mechanistic hierarchy (*D. ambrosioides* ≈ *N. oleander* >*A. nilotica* ≈ *C. procera* >*M. oleifera* >*L. inermis*) and supply a biochemical framework for designing botanical mixtures that combine rapid neurotoxic knockdown with sustained metabolic suppression. Despite the translational limitations posed by *N. oleander* toxicity, the integrated evidence supports the use of phenolic- and saponin-rich extracts as safer, multifunctional alternatives for stored-grain protection [58, 62, 64].

Building on these findings, future work will (i) perform comprehensive chromatographic profiling (HPLC, LC–MS) to pinpoint the principal bioactive compounds; (ii) fractionate extracts to validate the predicted mechanistic routes—such as midgut protease inhibition assays for tannin-rich fractions and membrane-leakage, mitochondrial, or in-vivo ROS analyses for saponin- or quinone-enriched fractions; (iii) benchmark these biochemical signatures against conventional insecticidal protectants; and (iv) develop safer formulations, including micro-encapsulated or low-dose analogs, with toxicity and food-safety evaluation under realistic storage conditions. These next steps will enable a mechanistic–applied bridge from laboratory findings to sustainable pest-management strategies based on locally accessible plant resources. 

## Supplementary Information

Supplementary Material 1.

## Data Availability

The datasets generated during and/or analyzed during the current study are available from the corresponding author upon reasonable request.
