# Supplementary material for: Phytochemistry and insecticidal activity of six local plant extracts against Sitotroga Cerealella (Oliv.) (Lepidoptera: Gelechiidae) in stored wheat
Source: BMC Plant Biol. 2025 Dec 29;26:165. doi: 10.1186/s12870-025-07879-8 (PMC12849392; doi:10.1186/s12870-025-07879-8)
Supplement: Supplementary file 1 — Supplementary Material 1. [file 12870_2025_7879_MOESM1_ESM.docx]

**Phytochemistry and Insecticidal Activity of Six Local Plant Extracts against *Sitotroga cerealella* (Oliv.) (Lepidoptera: Gelechiidae) in Stored Wheat.**

**Supplementary data**

**Table S1.** Physical characteristics of the methanolic extracts from the tested plant species.

| **Extracts** | **Color Before Extraction** | **pH** | **Color After Extraction** | **Texture** |
| --- | --- | --- | --- | --- |
| *Dysphania ambrosioides* | Light green | 5.48 | Dark green | Powder |
| *Calotropis procera* | Dark green | 4.96 | Dark green | Powder |
| *Moringa oleifera* | Dark green | 5.76 | Olive color | Gummy |
| *Nerium oleander* | Light green | 3.67 | Dark green | Waxy thick |
| *Lawsonia inermis* | Light red | 4.28 | Reddish brown | Waxy thick |
| *Acacia nilotica* | Light yellow | 4.34 | Shiny yellow | Powder |

**Table S2.** Qualitative screening of phytochemical constituents in *Dysphania ambrosioides*, *Calotropis procera*, *Moringa oleifera*, *Nerium oleander*, *Lawsonia inermis*, and *Acacia nilotica*.

| **Phytochemical** | ***Dysphania ambrosioides*** | ***Calotropis procera*** | ***Moringa oleifera*** | ***Nerium oleander*** | ***Lawsonia inermis*** | ***Acacia nilotica*** |
| --- | --- | --- | --- | --- | --- | --- |
| Alkaloids | – | – | – | **+** | – | – |
| Flavonoids | **+** | **+** | ++ | **+** | + | + |
| Tannins | – | **+** | +++ | **+++** | – | +++ |
| Steroids | **+** | **++** | ++ | **++** | + | + |
| Terpenoids | – | – | + | **+** | – | + |
| Phenols | **+** | **+** | ++ | **+** | ++ | +++ |
| Glycosides | **+** | **+** | + | **+** | + | + |
| Saponins | **+** | **++** | +++ | **+++** | +++ | +++ |
| Carbohydrates | **+** | **+** | + | **++** | +++ | + |
| Proteins | **++** | **++** | ++ | **+++** | + | +++ |

(-) Absent; (+) Low presence; (++) Moderate presence; (+++) High presence.

**Table S3.** Lethal times of methanolic *Dysphania ambrosioides*, *Calotropis procera*, *Moringa oleifera*, *Nerium oleander*, *Lawsonia inermis*, and *Acacia nilotica* extracts at 1, 3, 5, and 10 mg g^-1^ against *Sitotroga cerealella* adults. Values are lethal times (LT_50_ and LT_90_, days) with 95 % confidence limits in parentheses.

| **Extracts** | **Parameter** | **1 mg g⁻¹** | **3 mg g⁻¹** | **5 mg g⁻¹** | **10 mg g⁻¹** |
| --- | --- | --- | --- | --- | --- |
| *Dysphania ambrosioides* | LT_50_ (days)  (Upper- Lower) | 1.20 (0.75-1.52) | 0.68 (0.23-0.99) | 0.56(0.10-0.90) | 0.39 (0.03-0.73) |
|  | LT_90_ (days)  (Upper- Lower) | 8.31 (4.85-37.0) | 5.09 (3.23-18.2) | 5.34 (3.30-29.4) | 3.73(2.54-15.3) |
|  | Slope ±SE | 1.52 ±0.40 | 1.47± 0.40 | 1.31±0.4 | 1.32±0.42 |
| *Calotropis procera* | LT_50_ (days)  (Upper- Lower) | 1.61 (1.31-1.89) | 1.18(0.79-1.46) | 0.71(0.14-1.07) | 0.47(0.08-0.79) |
|  | LT_90_ (days)  (Upper- Lower) | 6.87 (4.66-15.5) | 6.51(4.24-18.3) | 8.19(4.33-14.6) | 3.79(2.62-12.3) |
|  | Slope ±SE | 2.02±0.38 | 1.72±0.38 | 1.20±0.39 | 1.42±0.42 |
| *Moringa oleifera* | LT_50_ (days)  (Upper- Lower) | 1.24(0.59-1.65) | 0.76(0.22-1.11) | 0.72(0.28-1.02) | 0.62(0.21-0.92) |
|  | LT_90_ (days)  (Upper- Lower) | 15.0(6.48-48.1) | 7.38(4.17-17.5) | 4.99(3.32-15.8) | 4.19(2.91-11.5) |
|  | Slope ±SE | 1.18±0.37 | 1.29±0.38 | 1.52±0.39 | 1.55±0.41 |
| *Nerium oleander* | LT_50_ (days)  (Upper- Lower) | 1.53(1.15-1.88) | 1.08(0.43- 1.47) | 0.50(0.09-0.82) | 0.38(0.05-0.69) |
|  | LT_90_ (days)  (Upper- Lower) | 9.30(5.43-36.8) | 12.9(5.89-36.2) | 4.10(2.78-14.4) | 2.82(2.09-6.64) |
|  | Slope ±SE | 1.63±0.37 | 1.18±0.37 | 1.40±41 | 1.48±0.44 |
| *Lawsonia inermis* | LT_50_ (days)  (Upper- Lower) | 2.54(2.20-3.13) | 2.06(1.76-2.50) | 1.04(0.55-1.36) | 0.47(0.06-0.80) |
|  | LT_90_ (days)  (Upper- Lower) | 8.26(5.68-16.8) | 8.31(5.46-19.6) | 7.87(4.58-38.9) | 4.30(2.84-18.8) |
|  | Slope ±SE | 2.50±0.41 | 2.12±0.38 | 1.46±0.38 | 1.33±0.41 |
| *Acacia nilotica* | LT_50_ (days)  (Upper- Lower) | 3.27(2.37-9.30) | 1.03(0.47-1.37) | 0.84(0.56-1.05) | 0.54(0.12-0.85) |
|  | LT_90_ (days)  (Upper- Lower) | 38.5(11.7-64.1) | 9.43(5.01-19.8) | 4.30(2.89-15.2) | 3.01(2.43-4.47) |
|  | Slope ±SE | 1.19±0.38 | 1.33±0.37 | 1.42±0.40 | 2.32±0.42 |
| Control | **NE**^*^ | | | | |

*NE: LT values could not be estimated because control mortality never reached 50% within 72 h under solvent-only conditions; hence slope is not applicable.

**Table S4.** Two-Way ANOVA data (Arcsine Square Root Transformed Data) for emergence reduction (%) of *Sitotroga cerealella*.

| **Source** | **df** | **Adj SS** | **Adj MS** | **F-value** | **P-value** |
| --- | --- | --- | --- | --- | --- |
| **Extracts** | 5 | 0.265 | 0.053 | 60.03 | <0.001 |
| **Concentration** | 3 | 2.659 | 0.886 | 1003.25 | <0.001 |
| **Extracts × Conc.** | 15 | 0.071 | 0.005 | 5.38 | <0.001 |
| **Error** | 72 | 0.064 | 0.001 |  |  |
| **Total** | 96 | 126.278 |  |  |  |
| **Corrected Total** | 95 | 3.059 |  |  |  |

**Table S5.** Two-Way ANOVA for grain weight loss (%) of wheat treated by the plant extracts.

| **Source** | **df** | **Adj SS** | **Adj MS** | **F-value** | **P-value** |
| --- | --- | --- | --- | --- | --- |
| **Extracts** | 5 | 83.662 | 16.732 | 8.86 | <0.001 |
| **Concentration** | 3 | 411.529 | 137.176 | 72.59 | <0.001 |
| **Extracts × Conc.** | 15 | 98.425 | 6.562 | 3.47 | <0.001 |
| **Error** | 75 | 141.725 | 1.890 |  |  |
| **Total** | 100 | 6797.279 |  |  |  |
| **Corrected Total** | 99 | 3196.799 |  |  |  |

**Table S6.** Two-Way ANOVA for insect-damaged grains (%) of wheat grain treated by the plant extracts.

| **Source** | **df** | **Adj SS** | **Adj MS** | **F-value** | **P-value** |
| --- | --- | --- | --- | --- | --- |
| **Extracts** | 5 | 6944.21 | 1388.84 | 240.01 | <0.001 |
| **Concentration** | 3 | 17088.13 | 5696.04 | 984.34 | <0.001 |
| **Extracts × Conc.** | 15 | 6424.63 | 428.31 | 74.02 | <0.001 |
| **Error** | 75 | 434.00 | 5.79 |  |  |
| **Total** | 99 | 110298.0 |  |  |  |
